# A new *Dictyostelium* prestalk cell sub-type

**DOI:** 10.1016/j.ydbio.2009.12.045

**Published:** 2010-03-15

**Authors:** Yoko Yamada, Robert R. Kay, Gareth Bloomfield, Susan Ross, Alasdair Ivens, Jeffrey G. Williams

**Affiliations:** a School of Life Sciences, University of Dundee, Dow St., Dundee DD3 5EH, UK; b Wellcome Trust Sanger Institute, Hinxton, UK; c MRC Laboratory of Molecular Biology, Hills Road, Cambridge, CB2 2QH, UK

**Keywords:** *Dictyostelium*, DimB, MybE, DIF-1, Cell type differentiation, Prestalk

## Abstract

The mature fruiting body of *Dictyostelium* consists of stalk and spore cells but its construction, and the migration of the preceding slug stage, requires a number of specialized sub-types of prestalk cell whose nature and function are not well understood. The prototypic prestalk-specific gene, *ecmA*, is inducible by the polyketide DIF-1 in a monolayer assay and requires the DimB and MybE transcription factors for full inducibility. We perform genome-wide microarray analyses, on parental, *mybE*− and *dimB*− cells, and identify many additional genes that depend on MybE and DimB for their DIF-1 inducibility. Surprisingly, an even larger number of genes are only DIF inducible in *mybE*− cells, some genes are only inducible in *DimB*− cells and some are inducible when either transcription factor is absent. Thus in assay conditions where MybE and DimB function as inducers of *ecmA* these genes fall under negative control by the same two transcription factors. We have studied in detail *rtaA*, one of the MybE and DimB repressed genes. One especially enigmatic group of prestalk cells is the anterior-like cells (ALCs), which exist intermingled with prespore cells in the slug. A promoter fusion reporter gene, rtaA:gal^u^, is expressed in a subset of the ALCs that is distinct from the ALC population detected by a reporter construct containing *ecmA* and *ecmB* promoter fragments. At culmination, when the ALC sort out from the prespore cells and differentiate to form three ancillary stalk cell structures: the upper cup, the lower cup and the outer basal disk, the rtaA:gal^u^ expressing cells preferentially populate the upper cup region. This fact, and their virtual absence from the anterior and posterior regions of the slug, identifies them as a new prestalk sub-type: the pstU cells. PstU cell differentiation is, as expected, increased in a *dimB*− mutant during normal development but, surprisingly, they differentiate normally in a mutant lacking DIF. Thus genetic removal of MybE or DimB reveals an alternate DIF-1 activation pathway, for pstU differentiation, that functions under monolayer assay conditions but that is not essential during multicellular development.

## Introduction

Differentiation in higher eukaryotes generally involves a cascade of transcription factor expression and activation that directs a cell within a particular lineage down its developmental pathway; each step along the way sets up the correct conditions for the following step. In *Dictyostelium* the situation is ostensibly simpler; cells first divide to form a field of cells that differentiate without the need for further cell division and there are only two ultimate cell fates: differentiation as a stalk cell or as a spore cell. The prestalk cell population is, however, divisible into discrete sub-types; pstA cells are located in the anterior 10% of slug length, pstO cells are located in a band immediately behind them and the ALCs lie within the prespore zone (Early et al., 1993). ALCs share characteristics with the anteriorly located prestalk cells and they are believed to have roles in slug migration and during final fruiting body formation. They show distinct movement patterns at culmination, when they come to form three ancillary structures: the upper cup, lower cup and outer basal disk (Sternfeld and David, 1982; Jermyn et al., 1996). The ALCs were originally identified by staining with vital dyes such as neutral red and significant advances in understanding their function has been made by marking them in this way, e.g. the demonstration that the upper cup behaves like a motor that lifts the spore head up the stalk (Sternfeld, 1998). However, for many purposes, markers of gene expression are preferable to vital dyes and they provide the evidence for multiple prestalk and ALC sub-types.

Differentiation into stalk cells is inducible by DIF-1 (henceforth termed DIF), a chlorinated hexaphenone that is produced by the prespore cells (Kay et al., 1999; Kay and Thompson, 2001; Thompson and Kay, 2000a). DIF rapidly activates transcription of the *ecmA* gene, a marker of prestalk differentiation (Williams et al., 1987). *ecmA* is expressed in pstA cells, pstO cells and a large subset of the ALCs (Early et al., 1993). *ecmA* expression in the pstA region is directed by cap-site proximal promoter sequences (termed the ecmA promoter region) while expression in the pstO region is directed by cap-site distal promoter sequences (termed the ecmO promoter region). A very high proportion of the ALCs express ecmO:gal and therefore resemble pstO cells (hence we term them the pstO-ALCs) but very few ALCs express ecmA:gal. Another important difference is that ecmO:gal expression is DIF-dependent while ecmA:gal expression is not (Thompson and Kay, 2000b).

Three transcription factors have been implicated in *ecmA* regulation: two bZIP proteins, DimA and DimB, and a single Myb domain protein, MybE (Thompson et al., 2004; Huang et al., 2006; Zhukovskaya et al., 2006; Fukuzawa et al., 2006). In vitro mapping experiments using recombinant proteins identified two DimB binding sites and two MybE binding sites within the ecmO promoter region (Zhukovskaya et al., 2006). DimB accumulates in the nucleus after DIF addition and ChIP analysis showed that DIF induction causes DimB to bind to the promoter of the *ecmA* gene in vivo. In a monolayer assay, measuring *ecmA* expression, the *dimA* null (dimA−), *dimB* null (*dimB*−) and *mybE* null (*mybE*−) strains are insensitive to DIF induction. At the slug stage the *mybE*− strain shows no significant expression of ecmAO:gal (lacZ under the transcriptional control of the complete promoter of the *ecmA* gene) in the pstO and pstO-ALCs. However, the situation for DimB is more complex; in Ax2 derived strains DimB functions as a repressor of *ecmA* gene transcription that acts selectively in the core of the pstA and pst O regions (Zhukovskaya et al., 2006) but in Ax4 it functions as an activator in pstO cells (Huang et al., 2006).

Although the *mybE* null strain shows no expression of ecmAO:gal in pstO or pstO-ALCs, staining with neutral red, a general marker of prestalk differentiation, indicates that there is a pstO region and that there are ALCs (Fukuzawa et al., 2006). Thus the effect of the *mybE* null mutation is to prevent individual gene transcription events within the pstO cell sub-type rather than to ablate the entire tissue. In contrast, null mutants in DimA, DimB and two DIF biosynthesis genes all show a much-reduced number of outer basal disk cells (Keller and Thompson, 2008; Saito et al., 2008). The outer basal disk derives from a coherent mass of ALCs, the pstB cells, located next to the substratum within the anterior of the prespore region (Dormann et al., 1996; Jermyn et al., 1996). PstB cells express the *ecmA* gene at a low level and a closely related gene, *ecmB*, at a much higher relative level. *ecmB* is also induced by DIF in the monolayer assay but more slowly than *ecmA* and with opposite sensitivities to exogenous extracellular cAMP; *ecmA* expression is stimulated by cAMP addition while *ecmB* expression is repressed (Williams et al., 1987; Berks and Kay, 1990).

*ecmA* expression depends upon DimB and MybE for DIF induction in a monolayer assay system but how general is this mode of DIF regulation? To answer this question, we performed array analysis of parental, DimB and MybE null cells in a DIF induction assay. This yielded many genes that show a similar DimB and MybE dependency as *ecmA* but revealed an even larger group of genes with the opposite dependency. One of these genes, *rtaA*, was characterized further and it defines a new ALC sub-type.

## Materials and methods

### Development and immuno-staining

Parental Ax2 (Gerisch isolate), *dimB*− and *mybE*− cells (dictyBase Stock Centre accession numbers DBS0235901 and DBS0236572) were treated with cAMP in monolayer in stalk medium (10 mM MES-KOH pH 6.2, 10 mM KCl, 2 mM NaCl, 1 mM CaCl_2_) containing 4 mM cyclic-AMP, 40 mM cerulenin (Sigma-Aldrich Company Ltd., Dorset, UK), 100 μg/ml streptomycin sulfate, at a density of 2.5 × 10^6^/ml and 2.5 × 10^7^ cells in a 9 cm dish for 9 h to render them DIF competent. They were then exposed to 100 nM DIF or solvent (0.1% ethanol) and incubated for a further 1 h. For normal development cells were plated on JA filters (Millipore, Watford, UK) sitting on 1.5% water agar plates, under overhead light to obtain culminants or dim unidirectional light to obtain migrating slugs. Structures were fixed with 50% methanol and then with 100% methanol. After rehydration, the cells were stained with mouse anti-β-gal monoclonal antibody (Cell Signalling Technology, Danvers MA, USA) and then with Alexa 594 conjugated anti-mouse antibody (Molecular Probes/Invitrogen, Paisley, UK) in PBS containing 5% BSA. For double staining, rabbit anti-β-glucuronidase antibody (Molecular Probes) and Alexa 488 conjugated anti-rabbit antibody (Molecular Probes) were also included.

### Array analysis

The array bears 9247 PCR products derived from *D. discoideum* ORFs, all printed in duplicate (Bloomfield et al., 2008). They non-redundantly cover 8579 predicted genes out of the recently estimated total of ∼ 10,300 different genes (Olsen, 2005), giving an ∼ 83% coverage. The arrays were hybridized and analyzed as described previously (Bloomfield et al., 2008). The raw and normalized data have been stored in the ArrayExpress database under the accession E-TABM-804.

### q-PCR and RT-PCR

Total RNA was prepared and treated with DNase using an RNeasy mini kit (Qiagen, West Sussex, UK). cDNA was synthesized with ImProm-II Reverse Transcription System (Promega, Southampton, UK), and analyzed for gene expression by q-PCR using iQ SYBR Green Supermix (Bio-Rad, Hemel Hemstead, UK). Expression was normalized to the constitutively transcribed gene IG7. The following primers were used:

*rtaA*:
  5′-TCGTGTTGCAGAATATGCAGGC-3′
  5′-TACGAAACCTGGGTGGAATGG-3′,
*Ig7*:
  5′-TTACATTTATTAGACCCGAAACCAAGCG-3′
  5′-AACAGCTATCACCAAGCTTGATTAGCC-3′

RT-PCR was performed using Super-Script One-Step RT-PCR with Platinum Taq (Molecular Probes).

### Construction of an rtaA promoter fusion

A genomic fragment, encompassing 1.1 kb upstream and including the first 33 nt of the coding region, of the *rtaA* gene was amplified by PCR using primers:

5′-TCTAGACGTTTCAGGTGAAACCTTAG-3′
5′-AGATCTGAGGTCGGCTAAATTTACCG-3′.

The product was cloned with Xba I and Bgl II to make a translational fusion to an ile-Gal reporter gene (Detterbeck et al., 1994).

## Results

### Expression-profiling of parental and mutant cells

**Fig. 1:**
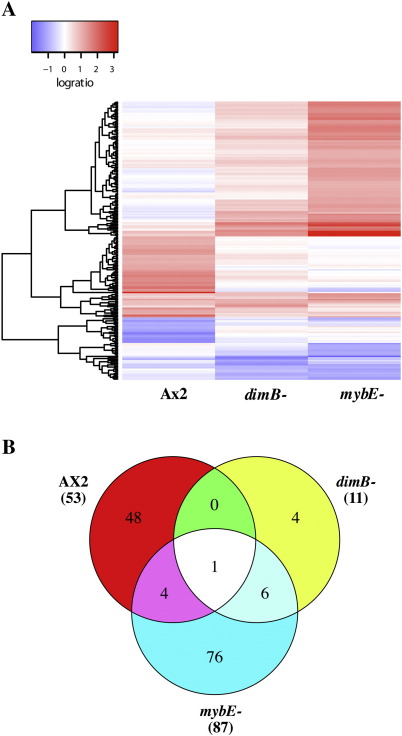
Array analysis of Ax2, *dimB*− and *mybE*− cells induced by DIF in a monolayer system. Ax2, *dimB*− and *mybE*− cells were induced by DIF in monolayer assay and gene expression was analyzed on a microarray. A) A HEAT diagram of the response to DIF. B) The numbers of genes up-regulated by DIF-1 more than 2-fold in each strain are shown in a Venn diagram. Monolayer medium included 4 mM cyclic-AMP and cells were stimulated with 100 nM DIF-1, or vehicle, for 1 h starting at 9 h of starvation (Ax2 and *mybE*−) or 10 h (*dimB*−, which develops more slowly).

Ax2, *dimB*− and *mybE*− cells developing in monolayer conditions with cAMP were exposed to DIF or left untreated and incubated for 1 h. RNA was extracted and array analysis was performed using genomic PCR products giving an approximate 83% coverage of the predicted set of 10,300 genes (Olsen, 2005; Bloomfield et al., 2008). The ratio of expression levels for DIF-treated and non DIF-treated cells is presented in Fig. 1A in the form of a heat map. Red bars indicate positive ratios (an induction by DIF) while blue bars indicate negative ratios (repression by DIF). There are many differences between the parental and mutant heat maps but the *mybE*− and *dimB*− mutants are more similar to each other than to the parental strain. This holds true for both the DIF-activated and DIF-repressed genes. It suggests that many DIF-regulated genes are similarly affected by the absence of MybE and by the absence of DimB.

Numerical analysis shows that 20 genes are at least two-fold repressed when Ax2 cells are exposed to DIF but are not repressed in *mybE*− or *dimB*− cells. Another 18 genes are two-fold repressed in *mybE*− cells but not in Ax2 cells. Since very little is known about repression of gene expression by DIF, we concentrated further effort on those genes that are up-regulated by at least two-fold upon induction with DIF (Supplementary Table S1). These array results are presented in the form of a Venn diagram in Fig. 1B. The absolute numbers should be treated with caution, because there was a dropout rate of 40% when 20 individual genes from various classes were selected for confirmation by PCR. Data for the 12 that scored positive in both array and PCR assay are shown in Supplementary Figs. S1 and S2.

One major up-regulated class is the 48 genes (red segment in Fig. 1B) that are only inducible in Ax2 cells. They, like *ecmA*, depend upon both MybE and DimB for their expression. The list of genes induced only in the parental strain includes both *ecmA* and *ecmB*. This is as expected, because *ecmB* is very closely related to *ecmA* in its protein structure and gene regulation. Because *ecmA* and *ecmB* are included in the list, analysis of the 48 gene products induced in Ax2 shows a highly significant GO term enrichment for extracellular matrix proteins (for term GO:0005201, extracellular matrix structural constituent, the *P*-value is 3.21E–02); no other GO term was significantly enriched in this group of genes. To our surprise, the largest class of DIF-induced genes is the 76 genes (blue segment) that are inducible in *mybE*− cells but not in Ax2 cells or *dimB*− cells (Fig. 1B). A smaller number, 4 genes (yellow segment), are inducible only in *dimB*− cells and 6 genes (pale blue segment) are inducible when either of the two transcription factors is absent. GO term analysis of genes induced in these strains did not show any grouping with a significant enrichment (data not shown).

### Promoter analysis of the MybE dependent DIF inducible genes

Two binding sites for MybE have been identified within the *ecmA* promoter and a third MybE binding site has been identified within the promoter of the *mrrA* gene (Fukuzawa et al., 2006; Tsujioka et al., 2007). The consensus of the three sites is A-A-C-a/t-G-T-T. We searched for this consensus within the set of 48 promoters that are, like *ecmA*, dependent upon MybE and DimB for DIF inducibility (Fig. 1B) normalizing against the total promoter set, analyzed using the same consensus sequence. In the set of 13,562 total promoter sequences there are 1553 sequences (11%) with at least one predicted MybE site. Among the 48 *mybE* dependent promoter sequences there are seven (15%) with at least one MybE site. The enrichment of MybE sites in the MybE dependent promoters is therefore, at best, very small.

The DimB binding site consensus, A-c/a-A-C-a/c-t/a-C-A, is based on just two sequences (Zhukovskaya et al., 2006). It is much less well defined than the MybE consensus; as evidenced by the presence of alternative bases at three positions and the fact that approximately 50% of all promoters have at least one copy of the sequence. DimB could, in principle, gain specificity by interacting functionally with MybE. The MybE analysis was performed using Genomatix software (Systat Software Inc.) that allows simultaneous scanning for different consensus binding sites with specified mutual separations. Such an analysis was performed for the MybE and the DimB consensus, specifying a separation equal to or less than 100 nucleotides; this was based upon the approximate separations found for these sites in the ecmO promoter. In the total promoter set this configuration occurs in 378 different promoters (2.8%) while in the selected set of 48 promoters the configuration occurs in only two promoters (4%); predictably, these are the promoters of the *ecmA* and *ecmB* genes. So again any enrichment in “new” target genes is very slight.

### Detailed analysis of rtaA gene expression

**Fig. 2:**
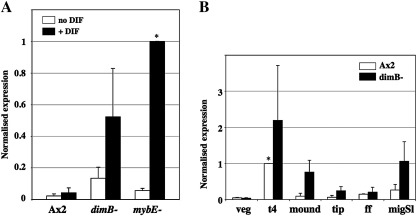
Quantitative analysis of *rtaA* expression in Ax2, *dimB*− and *mybE*− cells. A. AX2, *dimB*− and *mybE*− cells were cultured in monolayer condition with or without DIF as described in the Materials and methods and *rtaA* expression analyzed by q-PCR. Normalized expression in cells without DIF (open bars) and with DIF (black bars) is shown. B. AX2 (open bars) and *dimB*− (black bars) cells were developed to the indicated stages and analyzed for expression of *rtaA*. ff; first finger, migSl; migrating slug. In both A and B, expression is normalized to the sample marked *.

We next focused on one of the genes from the newly identified class that is negatively regulated by DimB and MybE (the pale blue segment in Fig. 1B). The selected gene, *rtaA*, (DDB_G0271852) was judged, from both the array and RT-PCR analyses, to be DIF inducible in MybE null cells and DimB null cells but not in parental cells (Supplemental Fig. S2). We first made a more quantitative determination of its DIF inducibility using q-PCR (Fig. 2A). In parental cells *rtaA* is DIF non-inducible but in *dimB* null cells it is approximately four-fold inducible and in *mybE* null cells it is approximately ten-fold inducible. A developmental time course of *rtaA* expression was constructed, also by q-PCR (Fig. 2B). This revealed an early peak of expression at around 4 h of development then an abrupt fall, followed by a rise in concentration in migrating slugs. There is a higher level of accumulation of *rtaA* mRNA in *dimB* null cells at all stages of development (Fig. 2B). We elected not to perform a similar quantitative analysis for the *mybE*− strain, because its developmental behavior is so different from the parent as to make a comparison meaningless (Fukuzawa et al., 2006).

### Determination of the expression pattern of an rtaA promoter:gal fusion

In order to identify cells expressing *rtaA* we created a lacZ fusion with *rtaA* upstream promoter elements. *rtaA* lies just over 1 kb downstream of a gene that is transcribed in the same direction as *rtaA* itself. The 1.1 kb of intervening, presumptive *rtaA* promoter sequence was cloned upstream of lacZ to yield rtaA:gal. However, the staining patterns that were obtained with this construct were variable from experiment to experiment. We reasoned that this might be due to β-galactosidase protein residual from the major *rtaA* expression pulse that occurs in early development (Fig. 2B). Hence we constructed an unstable reporter fusion. This construct rtaA:gal^u^ is similar in structure to rtaA:gal, except that it encodes a mutant form of β-galactosidase that is processed in the cell to reveal an ile residue at the N terminus. Such a protein has a much lower half-life than the parental form of the protein (Detterbeck et al., 1994).

**Fig. 3:**
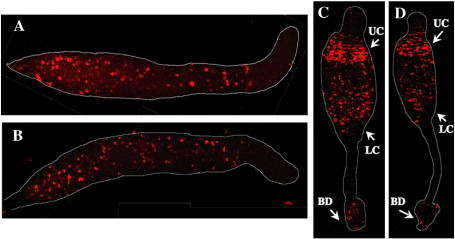
Analysis of the spatial expression pattern of *rtaA*. Ax2 cells carrying rtaA:gal^u^ were developed, fixed and stained with monoclonal anti-β-gal antibody and then with Alexa594 labeled anti-mouse antibody. *rtaA* is expressed in cells scattered in the prespore region of a slug, often with more expression in the posterior part of prespore region. There is little or no expression in the prestalk region. During culmination *rtaA* is expressed in the upper cup but much less so in the lower cup and the basal disk. Upper cup (UC), lower cup (LC), and basal disk (BD) are indicated with arrows.

We first attempted to use β-galactosidase staining to detect rtaA:gal^u^ expressing cells but, presumably because of the short half-life of the enzyme, the staining was too weak to be analyzed. We therefore turned to a more sensitive, immuno-histochemical detection method using a β-galactosidase antibody. At the slug stage *rtaA* is predominantly expressed in scattered cells, located throughout the prespore region (Fig. 3). At culmination a high proportion of rtaA:gal^u^ expressing cells accumulate in the region of the upper cup, many expressing cells remain scattered through the prespore region, there are a few expressing cells in the region of the basal disk and almost no expressing cells in the lower cup.

### Comparison of the rtaA expression pattern with that of commonly used prestalk markers

In order to localize the *rtaA* expressing cells relative to pstO and pstB cells, the two other DIF-regulated prestalk cell types, the rtaA:gal^u^ construct was stably co-transformed into cells along with ecmO:gus^nt^ or ecmB:gus^nt^. The latter two constructs encode β-glucuronidase and, to facilitate the identification of co-expressing cells, the reporter proteins are additionally marked by the presence of a nuclear localization sequence (Jermyn et al., 1996). We performed double immuno-staining using a red fluorochrome for β-galactosidase and a green fluorochrome for β-glucuronidase. Using this procedure cells expressing rtaA:gal^u^ but not the nuclear tagged gus reporter display a red cytoplasm and a dark region that is the presumptive nucleus. Cells that express the nuclear tagged gus reporter but not the gal reporter display green, presumptive nuclear staining. Cells that co-express the two reporters usually show a green nucleus in a red cell. However, the nucleus can occasionally appear yellow if, in that particular cell, the expression of the gus reporter is high and the position of the confocal section causes an overlay of cytosol and nucleus.

**Fig. 4:**
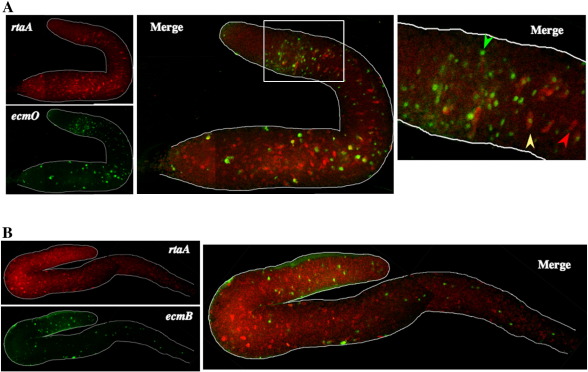
Double staining of *rtaA* with *ecmO* and *ecmB* expressing cells at the slug stage. Ax2 cells co-transformed with rtaA:gal^u^ and either ecmO:gus^nt^ (A) or ecmB:gus^nt^ (B) were developed, fixed and stained with mouse anti-β-gal antibody and rabbit anti-β-glucronidase antibody, and then with Alexa594 anti-mouse antibody and Alexa488 anti-rabbit antibody. Staining was observed under the confocal microscope and sections covering a few cell thicknesses were averaged. In A the region indicated by a white square is shown enlarged in the right panel. Arrows show cells expressing only *rtaA* (red), only ecmO (green), or both (yellow). In the pstO region, there are many cells that express ecmO but not *rtaA*. This is more apparent in the anterior part of the pstO region. Within the posterior half of the pstO region, some cells co-expressing *rtaA* are observed. The *ecmB* expressing cells rarely if ever show expression of *rtaA*.

In slugs ecmO:gus^nt^ detects the pstO population and the subset of ALCs that utilize ecmO promoter elements: i.e. the pstO-ALCs (Fig. 4A). The position of the prestalk boundary, defined by the band of ecmO:gus^nt^ expressing cells, confirms that the rtaA:gal^u^ expressing cells are predominantly confined to the prespore region, although there are some rtaA:gal^u^ expressing cells located towards the back of the pstO region. A few of these cells co-express rtaA:gal^u^ and ecmO:gus^nt^, e.g. the cell indicated with a yellow arrow in Fig. 4A. Examination of the prespore region shows that most of the ALCs that express ecmO:gus^nt^, i.e. the pstO-ALCs, do not co-express rtaA:gal^u^. Conversely, most of the ALCs that express rtaA:gal^u^ do not co-express ecmO:gus^nt^.

In slugs expressing ecmB:gus^nt^ staining cells are highly enriched in the prestalk region, with relatively few expressing cells in the prespore zone (Fig. 4B). The absence of double staining cells shows that the pstB-ALCs do not express rtaA:gal^u^.

### Analysis of culmination by double staining

**Fig. 5:**
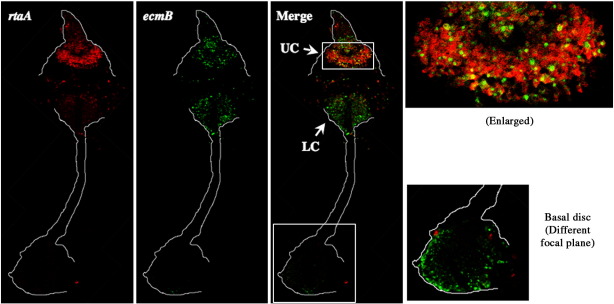
Double staining of *rtaA* and *ecmB* expressing cells at culmination. Ax2 cells co-transformed with rtaA:gal^u^ and ecmB:gus^nt^ were developed and analyzed for expression as in Fig. 4. An enlargement of the upper cup region is shown in the right top panel. The basal disk, at a different focal plane and also enlarged, is shown in the right bottom panel. The double staining shows that cells expressing *rtaA* are under-represented in the lower cup and basal disk. Analysis of many structures shows that there are many cells in the upper cup that express only *ecmB* or *rtaA* and about half the cells in this region express both *ecmB* and *rtaA*.

The above data show that there are at least three intermingled populations of ALCs within the prespore region of the slug: pstO-ALCs, pstB-ALCs and ALCs that express rtaA:gal^u^. The latter population qualifies as ALCs because (Fig. 3), at culmination most of the rtaA:gal^u^ expressing cells become localized to the upper cup region and in later culminants they acquire the flattened shape typical of cells about to become stalk cells, e.g. the red stained cells in the papilla in Fig. 3D. The upper cup was originally defined using *ecmB* as a marker so it was of importance to perform double staining at culmination with rtaA:gal^u^ and ecmB:gus^nt^. As expected, the ecmB:gus^nt^ expressing cells accumulate in the upper cup, lower cup and outer basal disk while the rtaA:gal^u^ expressing cells become highly enriched in the upper cup region (Fig. 5). There is a major increase in the number of ecmB expressing cells at culmination and many cells co-express the two markers. We estimate that about half of upper cup cells show co-expression but there are many that express only *ecmB* or *rtaA*. Transitorily therefore, at the mid-culminant stage, the upper cup is a mosaic composed of two prestalk cell sub-types.

### Analysis of *rtaA* spatial patterning in the *dimB*− strain

**Fig. 6:**
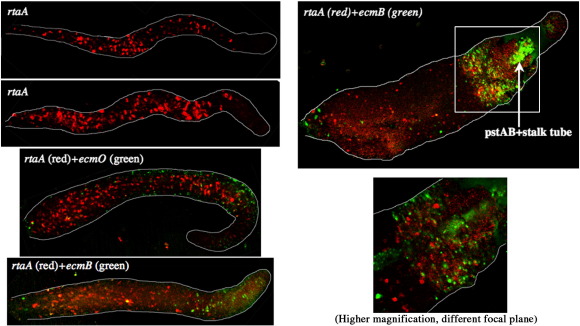
The spatial expression pattern of *rtaA* in *dimB* null cells. *dimB*− cells carrying the indicated marker genes were developed, fixed and stained as in Fig. 4. For double staining of slugs, confocal sections covering a few cell thicknesses were averaged. Double staining of *rtaA* and ecmO or *rtaA* and *ecmB* in the dimB null show no apparent increase of overlap in expression of the two genes compared to parental cells. Staining of culminants is also similar to the wild type; *rtaA* is expressed in the upper cup but not in the lower cup. This is an early culminant, hence the stalk tube is rudimentary and the pstAB core cells are visible. pstAB cells co-express *ecmA* and *ecmB* at a high level.

In order to determine whether the elevated *rtaA* gene expression in *dimB*− slugs (Fig. 2B) is accompanied by an altered spatial expression pattern, the *dimB*− strain was co-transformed with the rtaA:gal^u^ reporter and either the ecmB or the ecmO gus markers. At both the slug stage and at culmination the expression pattern was qualitatively similar to that observed in the parental strain (Fig. 6). In order to determine whether there is a quantitative change, in the number of cells expressing *rtaA*, we dissociated migrating slugs derived from Ax2 cells and *dimB*− cells transformed with rtaA:gal^u^. Two pools of each transformants were analyzed, with similar results, and the data for one pool, analyzed in three separate experiments, are presented in Table 1. There is an approximate 50% increase in the proportion of *rtaA* expressing cells in the *dimB* null but this cannot account for the approximate 3 to 4-fold increase in total *rtaA* expression in the mutant strain. Presumably, therefore there is a higher level of *rtaA* expression in individual *dimB* null mutant cells than in parental cells.

### Analysis of *rtaA* spatial patterning in a DIF-deficient strain

**Fig. 7:**
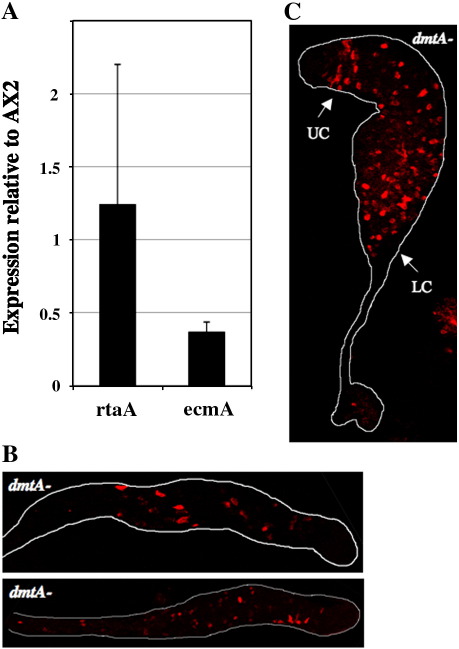
The spatial expression pattern of *rtaA* in a DIF-deficient mutant. A) Ax2 and *dmtA*− cells were developed to the slug stage and f *ecmA* and *rtaA* expression determined by q-PCR. Results are expressed as the level of expression in *dmtA*− slugs relative to the level in Ax2 slugs. B) and C) *dmtA*− cells were transformed with rtaA:gal^u^, developed to the slug stage (B) or mid-culminant stage (C), fixed and stained for β-galactosidase.

The *dmtA*− strain contains a disruption in the gene encoding the tranferrase that methylates the immediate precursor of DIF-1. The strain expresses *ecmA* in the pstA cells but not in pstO cells. We first quantitated total *ecmA* gene expression in mutant slug cells, relative to Ax2 parental slug cells, using q-PCR (Fig. 7A). As expected, given the absence of *ecmA* expression in pstO cells, *dmtA*− slugs are partially defective in total *ecmA* expression. Surprisingly, however, they display a quantitatively normal level of *rtaA* expression (Fig. 7A). The rtaA:gal^u^ reporter was transformed into the *dmtA*− strain and antibody staining of slugs and culminants confirms that *rtaA* is expressed normally (Figs. 7B and C).

## Discussion

We have identified 48 genes that are, like *ecmA*, DIF inducible in a monolayer assay and that require the activity of MybE and DimB for their expression. Apart from the two well-characterized DIF targets, the extracellular matrix protein-encoding genes *ecmA* and *ecmB*, the gene products do not show any statistically significant enrichment in a GO term analysis. Also, we are unable to discern any enrichment within their promoters for the known MybE and DimB binding sites. This could mean that the MybE and DimB requirement of these genes is indirect or, more simply, that the training set is too small and that we need a better definition of the DimB and MybE binding site consensus. The array analysis also revealed unexpected classes of genes that show radically different behavior from *ecmA* and *ecmB* with respect to DimB and MybE. A large group of genes become DIF inducible when *mybE* is genetically disrupted, a much smaller class become inducible when *dimB* is disrupted and an intermediate-sized class becomes inducible when either gene is disrupted.

We chose to focus on the third class. *rtaA*, the representative gene chosen for this family, is highly DIF inducible in *mybE*− cells, less strongly inducible in *dimB*− cells and non-inducible in Ax2 cells. The Rta1 family of proteins is ubiquitous in fungi, where they are sometimes associated with xenobiotic resistance (Mamente and Ghislain, 2009).They are predicted to contain multiple trans-membrane domains. *rtaA* is induced when phagocytosis is triggered (Sillo et al., 2008 ) and it will be of interest to determine if there is any relationship to its DIF inducibility. Analysis of *rtaA* expression during normal development revealed two rises, one in early development and the other at the slug stage. An rtaA:lacZ fusion gene is not expressed in pstA cells. It is expressed in a small proportion of the posteriorly located pstO cells and also in many of the ALCs. This is quite unlike the ecmAO promoter, which is active in all anterior prestalk cells and also in large numbers of ALCs. Double staining shows that, despite being intermingled, there is little overlap between the individual ALCs that express *rtaA* and the pstO-ALCs. The promoters of the genes encoding AmpA, Ga4, Ga5, PTP1, PTP2 and ERK1 are also active in the ALCs and are not selectively expressed in cells in the prestalk region (Casademunt et al., 2002; Gaskins et al., 1994; Hadwiger and Firtel, 1992; Hadwiger et al., 1996; Howard, 1992; Howard et al., 1994). However, in those cases where it is known cells expressing these other markers sort to both the upper and lower cups at culmination and a defining characteristic of rtaA:lacZ expressing cells is that they become highly enriched in the upper cup of the culminant. Because of the above features we deem them to be a novel prestalk sub-type and propose naming them as pstU cells.

The identification of a class of ALCs that populate the prespore region rather than the prestalk region and that move selectively to the upper cup at culmination raises a number of important issues. The existence of two classes of upper cup cells, those that express *rtaA* and those that express *ecmB*, implies a possible functional heterogeneity. The only function thus far ascribed to the upper cup is as a cellular motor that elevates the spore head up the stalk (Sternfeld, 1998). It will be of interest to know whether PstU cells participate in this process. Two additional major questions concern the signal that induces PstU cell differentiation and the way in which they come to selectively populate the prespore region. The latter question is difficult to answer but we have investigated the former issue genetically.

MybE and DimB are both essential for DIF induction of *ecmA* in monolayer systems and DIF causes DimB to move to the nucleus where it binds to the *ecmA* promoter. Moreover, a 22 nt fragment of *ecmA* promoter DNA that contains a MybE binding site is sufficient, when multimerized, to confer DIF inducible expression on a lacZ reporter (Fukuzawa et al., 2006). Thus there is, in the monolayer system, a strong case for a direct DIF-regulated signaling pathway involving MybE and DimB as activators. *rtaA*, in contrast, is not DIF inducible in parental cells but is inducible in *mybE*− and *dimB*− cells. Thus MybE and DimB are not required for the inducibility of the *rtaA*-like genes. This implies the existence of an alternate signaling pathway that uses a different transcriptional activator.

There is a separate DIF induction pathway that regulates the tyrosine phosphorylation of STATc but this operates by phosphorylating the PTP3 tyrosine phosphatase, so repressing its activity towards STATc (Araki et al., 2008). Hence it seems unlikely to interface with either of the two pathways postulated here. Therefore, there could be as many as three partially or wholly distinct DIF signalling pathways operative in the monolayer assay. However, it seems very doubtful that these same pathways function in the same ways during multicellular development, because: i) the fact that a DIF-deficient mutant expresses *rtaA* during multicellular development indicates that some other signaling molecule must be the biologically relevant inducer and ii) DimB acts as a strong negative regulator of *rtaA* in the monolayer assay system but is only a partial inhibitor of *rtaA* expression during normal development.

There are precedents for such incongruities between the monolayer assay and normal development. For example, DIF-induced expression of *ecmA* in a monolayer system is entirely dependent upon DimB but in an Ax2 parental background expression of *ecmA* is elevated in the dimB null strain. We should perhaps not be too surprised by these differences; cells within a multicellular milieu receive direct inputs, from their immediate neighbors and the extracellular matrix, and diffusible signals from distant sources. The monolayer assay is, therefore, an invaluable analytical tool for discovering new inducing factors and, as here identifying responsive genes and studying their associated signal transduction pathways. However, rather like mammalian tissue culture models, it acts as an imperfect mirror for the normal process.

## Figures and Tables

**Figure:**
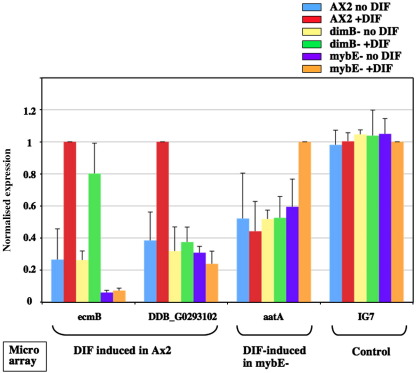


**Figure:**
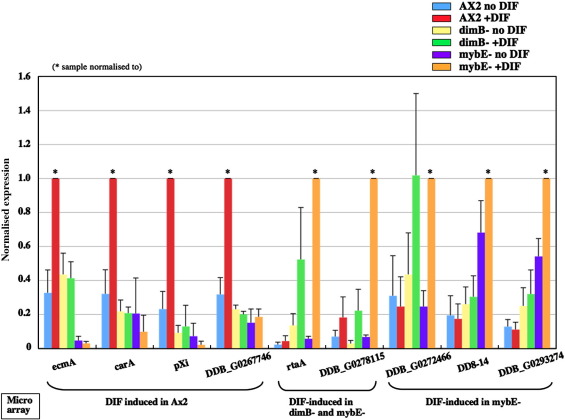


**Table 1:** A comparison of PstU differentiation in parental and *dimB*− cells.

% rtaA expressing	
Strain	cells
Ax2	9.0 ± 2.7
*dimB*−	13.9 ± 4.6

Migrating slugs formed by Ax2 and dimB null cells transformed with rtaA:gal^u^ were dissociated and fixed in 80% methanol. Cells were then stained with a mouse anti-β-gal antibody and Alexa594 conjugated anti-mouse antibody. The fraction of staining cells was determined by microscopic counting.
