# Supplementary material for: A new Dictyostelium prestalk cell sub-type
Source: Dev Biol. 2010 Mar 15;339(2-3):390–7. doi: 10.1016/j.ydbio.2009.12.045 (PMC2845816; doi:10.1016/j.ydbio.2009.12.045)
Supplement: Supplementary file 1 [file mmc1.doc]

**Table S1 The genes induced by DIF-1 more than two fold in parental and mutant cells**

| strain with upregulation | Gene name | DictybaseID | fold induction by DIF | Gene product |
| --- | --- | --- | --- | --- |
| Ax2 | DDB_G0274161 | DDB0167534 | 9.4 |  |
|  | DDB_G0276817 | DDB0168264 | 3.4 | K08H10.2A PROTEIN. 6/100 |
|  | DDB_G0292630 | DDB0184486 | 5.9 |  |
|  | DDB_G0292668 | DDB0184513 | 3.3 |  |
|  | DDB_G0287985 | DDB0187721 | 3.5 |  |
|  | DDB_G0288001 | DDB0187729 | 3.3 |  |
|  | DDB_G0288033 | DDB0187746 | 2.3 |  |
|  | DDB_G0288495 | DDB0187960 | 3.1 |  |
|  | DDB_G0289233 | DDB0188326 | 3.9 | Expressed protein. |
|  | DDB_G0289273 | DDB0188343 | 2.3 |  |
|  | DDB_G0289605 | DDB0188487 | 6.0 |  |
|  | DDB_G0267746 | DDB0189516 | 3.2 |  |
|  | DDB_G0268006 | DDB0189712 | 2.7 |  |
|  | tmem56C | DDB0191042 | 3.5 | TRAM, LAG1 and CLN8 homology domain-containing protein, TMEM56 family protein 3 |
|  | DDB_G0293102 | DDB0191783 | 2.9 |  |
|  | pter | DDB0191915 | 4.1 | phosphotriesterase-related protein |
|  | DDB_G0271104 | DDB0202898 | 3.2 |  |
|  | DDB_G0274677 | DDB0203229 | 7.1 |  |
|  | DDB_G0275967 | DDB0203533 | 2.1 | amidase family protein |
|  | DDB_G0271764 | DDB0203624 | 2.1 |  |
|  | DDB_G0282305 | DDB0204168 | 7.0 | Gp63 homolog. |
|  | ecmB | DDB0216219 | 3.3 | extracellular matrix protein ST310 |
|  | carA-2 | DDB0217181 | 3.2 | cAMP receptor 1 |
|  | DDB_G0273921 | DDB0217374 | 2.3 | zinc-containing alcohol dehydrogenase (ADH) |
|  | DDB_G0274031 | DDB0217428 | 2.4 | unknown |
|  | DDB_G0274921 | DDB0217548 | 2.6 | Similar to Homo sapiens (Human). BK1048E9.3 (Novel protein). |
|  | DDB_G0283591 | DDB0218508 | 6.7 |  |
|  | DDB_G0285779 | DDB0218739 | 4.4 |  |
|  | DDB_G0290897 | DDB0219617 | 6.7 |  |
|  | DDB_G0277539 | DDB0220004 | 3.9 | WEE1 family protein kinase, putative protein tyrosine kinase |
|  | ecmA | DDB0220137 | 2.8 | extracellular matrix protein ST430 |
|  | DDB_G0289713 | DDB0220682 | 5.3 | SAP DNA-binding domain-containing protein |
|  | DDB_G0271402 | DDB0229866 | 2.7 | protein kinase, TKL group, LISK family protein kinase |
|  | DDB_G0271682 | DDB0229871 | 2.7 | protein kinase, TKL group, SMAD/FHA domain-containing protein |
|  | mkcD | DDB0229913 | 2.1 | MKC subfamily protein kinase, protein kinase, STE group |
|  | DDB_G0283871 | DDB0229937 | 11.8 | unknown |
|  | gpaI | DDB0230128 | 3.6 | G-protein subunit alpha 9 |
|  | grlQ | DDB0304720 | 2.9 | G-protein-coupled receptor (GPCR) family 3 protein 16 |
|  | gtr3 | DDB0231974 | 4.1 | putative glycosyltransferase |
|  | CYP513C1 | DDB0232340 | 3.4 | cytochrome P450 family protein |
|  | pXi | DDB0232940 | 2.6 | CAMK1 family protein kinase |
|  | swp1 | DDB0233147 | 2.4 | oligosaccharyltransferase delta subunit, dolichyl-diphosphooligosaccharide-protein glycotransferase |
|  | DDB_G0287061 | DDB0233290 | 2.1 | Rab GTPase domain-containing protein |
|  | DDB_G0274795 | DDB0233894 | 2.5 | peptidase C19 family protein, ubiquitin-specific protease 48-like protein |
|  | DDB_G0278809 | DDB0233967 | 2.8 | centrosomal protein 148 kDa |
|  | DDB_G0288405 | DDB0235328 | 2.9 | RabGAP/TBC domain-containing protein, TLDc domain-containing protein |
|  | DDB_G0271348 | DDB0237737 | 3.1 | putative ubiquitin carboxyl-terminal hydrolase (UCH), peptidase C19 family protein |
|  | DDB_G0275959 | DDB0237825 | 2.7 | cellular retinaldehyde-binding/triple function domain-containing protein |
|  |  |  |  |  |
| dimB- | DDB_G0278333 | DDB0205381 | 2.5 | SppA. |
|  | DDB_G0278571 | DDB0205545 | 2.5 | hypoxia induced family protein |
|  | mfeB | DDB0214811 | 2.4 | hypothetical peroxisomal multifunctional enzyme 2 |
|  | osbJ | DDB0237798 | 2.8 | oxysterol binding family protein, member 10 |
|  |  |  |  |  |
| mybE- | DDB_G0274339 | DDB0167887 | 2.2 | acetyl-CoA C-acyltransferase, 3-ketoacyl-CoA thiolase |
|  | DDB_G0271920 | DDB0168559 | 2.9 | unknown |
|  | DDB_G0277461 | DDB0169262 | 9.7 |  |
|  | DDB_G0276291 | DDB0169426 | 3.2 |  |
|  | med14 | DDB0183801 | 4.3 | putative mediator complex subunit 14 |
|  | DDB_G0292764 | DDB0184550 | 2.4 |  |
|  | DDB_G0285447 | DDB0186498 | 4.8 | DUF829 family protein, putative transmembrane protein |
|  | DDB_G0285615 | DDB0186611 | 2.9 | unknown |
|  | DDB_G0286567 | DDB0187011 | 2.6 |  |
|  | DDB_G0288201 | DDB0187826 | 2.6 |  |
|  | DDB_G0289017 | DDB0188213 | 3.2 |  |
|  | adprh | DDB0188511 | 3.3 | ADP-ribosylarginine hydrolase |
|  | DDB_G0291083 | DDB0189226 | 2.5 |  |
|  | DDB_G0267494 | DDB0189323 | 5.6 |  |
|  | psiS | DDB0189707 | 3.5 | PA14 domain-containing protein |
|  | DDB_G0268002_ps | DDB0189709 | 2.9 | pseudogene |
|  | DDB_G0268208 | DDB0189867 | 3.9 |  |
|  | DDB_G0268846 | DDB0190081 | 2.2 |  |
|  | DDB_G0268848 | DDB0190082 | 2.4 | putative ATP binding protein |
|  | DDB_G0269588 | DDB0190384 | 2.5 | FadA6_3. |
|  | cshA | DDB0191110 | 2.3 | citrate synthase |
|  | pykA | DDB0191114 | 2.8 | pyridoxal kinase |
|  | abcG2 | DDB0191229 | 8.8 | ABC transporter G family protein |
|  | acoA | DDB0191408 | 2.4 | acyl-CoA oxidase |
|  | rbrA | DDB0191418 | 2.7 | ariadne-like ubiquitin ligase, C3HC4-type zinc finger-containing protein |
|  | vacA | DDB0191505 | 2.3 | vacuolin A, prohibitin domain-containing protein |
|  | DDB_G0291932 | DDB0191609 to5prime | 8.2 |  |
|  | DDB_G0293594 | DDB0192030 | 4.9 |  |
|  | mfeA | DDB0201628 | 2.8 | peroxisomal multifunctional enzyme MFE homolog |
|  | DDB_G0268318 | DDB0202120 | 2.6 |  |
|  | DDB_G0268932 | DDB0202285 | 8.4 |  |
|  | DDB_G0274663 | DDB0203213 | 2.5 | P90893 Putative serine protease F56F10.1 precursor (EC 3.4.-.-). |
|  | DDB_G0282341 | DDB0204191 | 3.2 |  |
|  | DDB_G0278123 | DDB0204442 | 4.4 |  |
|  | ech1 | DDB0205302 | 2.4 | enoyl Coenzyme A hydratase, Delta(3,5)-Delta(2,4)-dienoyl-CoA isomerase |
|  | DDB_G0278297 | DDB0205355 | 2.9 | metallophosphoesterase domain-containing protein |
|  | DDB_G0279429 | DDB0205761 | 2.3 |  |
|  | DDB_G0279935 | DDB0205870 | 2.3 |  |
|  | cnxA | DDB0215348 | 3.0 | calnexin |
|  | DDB_G0293274 | DDB0215559 | 2.9 |  |
|  | DDB_G0289787 | DDB0216125 | 3.1 |  |
|  | dymA | DDB0216177 | 2.4 | dynamin like protein |
|  | rsc5 | DDB0216218 | 2.2 | cellular retinaldehyde-binding/triple function domain-containing protein |
|  | lpd | DDB0216232 | 2.2 | dihydrolipoamide:NAD oxidoreductase, dihydrolipoamide dehydrogenase, glycine cleavage system L-protein |
|  | H2Bv2 | DDB0216304 | 2.1 | histone H2B domain-containing protein |
|  | psiE | DDB0216573 | 2.8 | PA14 domain-containing protein |
|  | DDB_G0272464 | DDB0217019 | 3.2 |  |
|  | DDB_G0272466 | DDB0217020 | 5.2 | short-chain dehydrogenase/reductase (SDR) family protein, glucose/ribitol dehydrogenase family protein |
|  | hspF-2 | DDB0217196 | 2.5 | heat shock protein Hsp20 domain-containing protein, putative alpha-crystallin-type heat shock protein |
|  | DDB_G0274831 | DDB0217501 | 2.4 | glycoside hydrolase family 24 protein, putative T4-like lysozyme |
|  | DDB_G0280739 | DDB0218244 | 7.7 |  |
|  | DDB_G0288469 | DDB0219344 | 3.3 |  |
|  | sgkC | DDB0220132 | 3.5 | sphingosine kinase related protein |
|  | aco1 | DDB0229908 | 3.6 | aconitase, putative iron regulatory protein |
|  | aatA | DDB0230092 | 2.7 | aspartate aminotransferase |
|  | mdhA | DDB0230186 | 2.9 | malate dehydrogenase |
|  | DDB_G0287735 | DDB0230204 | 2.8 | putative 5'-nucleotidase |
|  | fcsB | DDB0231574 | 3.1 | fatty acyl-CoA synthetase, long-chain-fatty-acid-CoA ligase |
|  | DDB_G0274661 | DDB0231658 | 2.9 | unknown |
|  | DD8-14 | DDB0231686 | 2.2 | AAA ATPase domain-containing protein |
|  | cycL | DDB0231778 | 3.7 | cyclin |
|  | DDB_G0283913 | DDB0232159 | 2.7 | heat shock protein Hsp20 domain-containing protein |
|  | DDB_G0276219 | DDB0233371 | 3.3 | putative transmembrane protein |
|  | DDB_G0276095 | DDB0233372 | 3.1 | unknown |
|  | DDB_G0276361 | DDB0233513 | 2.1 | unknown |
|  | DDB_G0272204 | DDB0233554 | 7.3 | Fis-type helix-turn-helix domain-containing protein |
|  | DDB_G0287741 | DDB0233834 | 2.2 | enoyl-CoA hydratase/isomerase domain-containing protein |
|  | DDB_G0267734 | DDB0233903 | 2.2 | putative SAM dependent methyltransferase |
|  | DDB_G0276321 | DDB0233947 | 2.3 | putative acetoacetyl-CoA synthetase, AMP-dependent synthetase and ligase domain-containing protein |
|  | DDB_G0275033 | DDB0234029 | 2.7 | unknown |
|  | DDB_G0270990 | DDB0234155 | 2.4 | putative acyl-CoA oxidase |
|  | DDB_G0271270 | DDB0237488 | 2.4 | beta-lactamase-like domain-containing protein |
|  | DDB_G0272728 | DDB0237647 | 3.1 | unknown |
|  | DDB_G0283911 | DDB0237822 | 2.9 | HSP20-like chaperone domain-containing protein |
|  | DDB_G0288011 | DDB0237960 | 2.4 | methyltransferase type 11 domain-containing protein |
|  |  |  |  |  |
| dimB- and mybE- (a) | DDB_G0271852 | DDB0168536 | 3.6 | RTA1 protein. |
|  | DDB_G0285177 | DDB0186377 | 2.6 |  |
|  | DDB_G0278115 | DDB0204431 | 10.8 | DUF829 family protein, putative transmembrane protein |
|  | DDB_G0276097 | DDB0233370 | 2.6 | putative transmembrane protein |
|  | gacZ | DDB0233848 | 5.2 | RhoGAP domain-containing protein, MYND-type zinc finger-containing protein |
|  | DDB_G0279791 | DDB0235170 | 2.3 | unknown |
|  |  |  |  |  |
| Ax2 and mybE- (b) | DDB_G0268974 | DDB0202320 | 15.1 |  |
|  | DDB_G0270240 | DDB0190904 | 5.7 |  |
|  | DDB_G0286689 | DDB0187097 | 4.4 | unknown |
|  | DDB_G0286801 | DDB0218850 | 8.2 |  |
|  |  |  |  |  |
| Ax2, dimB- and mybE- (c) | cutA | DDB0191386 | 2.3 | fatty acid elongase 3-ketoacyl-CoA synthase, long chain fatty acid elongase |
